# Large Scale Chemical Cross-linking Mass Spectrometry Perspectives

**DOI:** 10.4172/jpb.S2-001

**Published:** 2013-02-08

**Authors:** Boris L. Zybailov, Galina V. Glazko, Mihir Jaiswal, Kevin D. Raney

**Affiliations:** 1 Department of Biochemistry and Molecular Biology, University of Arkansas for Medical Sciences, Little Rock, AR, USA; 2 Department of Biomedical Informatics, University of Arkansas for Medical Sciences, Little Rock, AR, USA; 3 UALR/UAMS Joint Bioinformatics Program, University of Arkansas Little Rock, Little Rock, AR, USA

**Keywords:** Chemical cross linking, Mass spectrometry, Proteomics, Large-scale PPI

## Abstract

The spectacular heterogeneity of a complex protein mixture from biological samples becomes even more difficult to tackle when one’s attention is shifted towards different protein complex topologies, transient interactions, or localization of PPIs. Meticulous protein-by-protein affinity pull-downs and yeast-two-hybrid screens are the two approaches currently used to decipher proteome-wide interaction networks. Another method is to employ chemical cross-linking, which gives not only identities of interactors, but could also provide information on the sites of interactions and interaction interfaces. Despite significant advances in mass spectrometry instrumentation over the last decade, mapping Protein-Protein Interactions (PPIs) using chemical cross-linking remains time consuming and requires substantial expertise, even in the simplest of systems. While robust methodologies and software exist for the analysis of binary PPIs and also for the single protein structure refinement using cross-linking-derived constraints, undertaking a proteome-wide cross-linking study is highly complex. Difficulties include i) identifying cross-linkers of the right length and selectivity that could capture interactions of interest; ii) enrichment of the cross-linked species; iii) identification and validation of the cross-linked peptides and cross-linked sites.

In this review we examine existing literature aimed at the large-scale protein cross-linking and discuss possible paths for improvement. We also discuss short-length cross-linkers of broad specificity such as formaldehyde and diazirine-based photo-cross-linkers. These cross-linkers could potentially capture many types of interactions, without strict requirement for a particular amino-acid to be present at a given protein-protein interface. How these shortlength, broad specificity cross-linkers be applied to proteome-wide studies? We will suggest specific advances in methodology, instrumentation and software that are needed to make such a leap.

## Protein-Protein Interactions: Research Strategies

**Figure 1:**
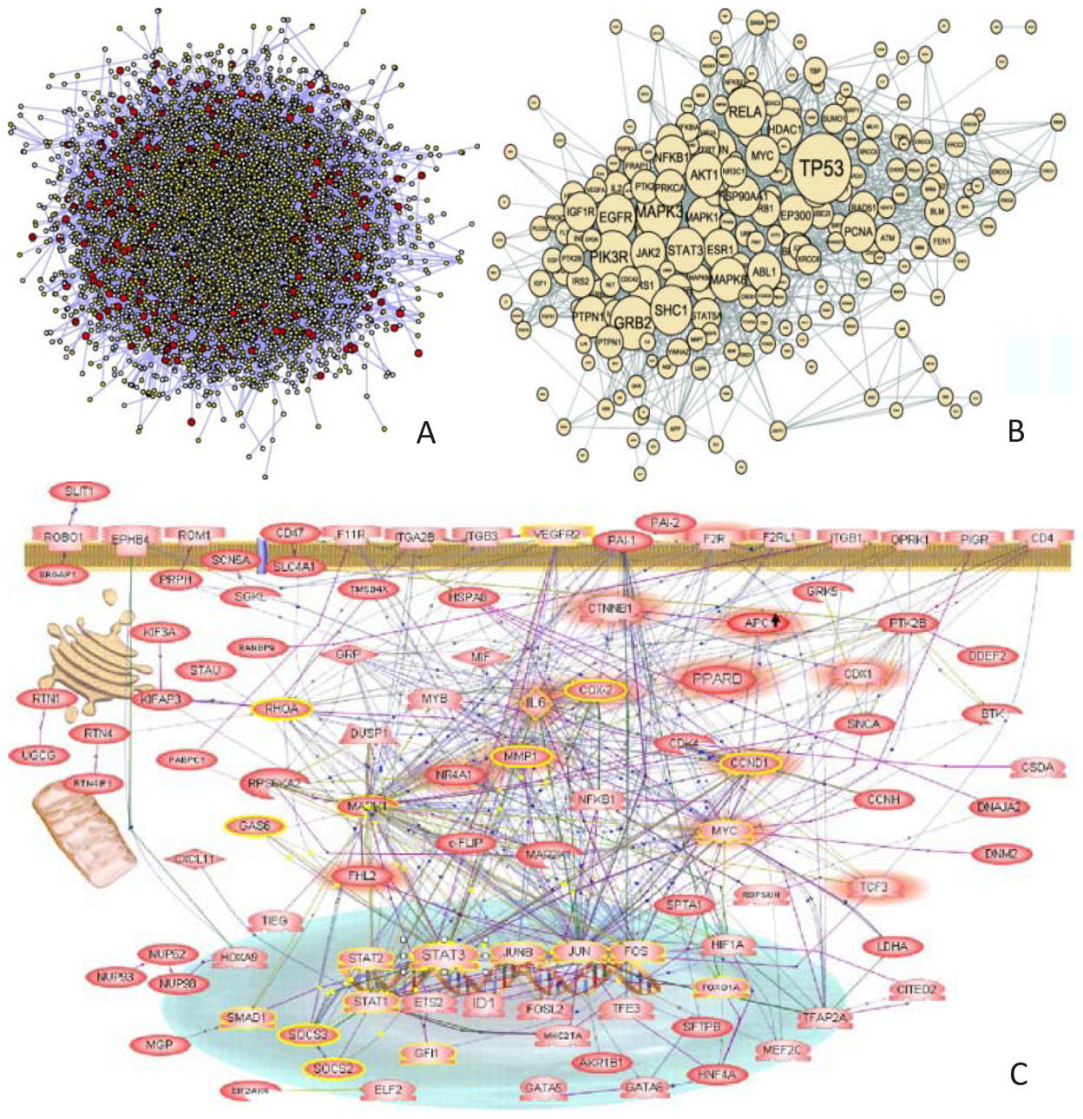
Examples of Biological Networks A) Human Protein-Protein interaction network, taken from [117], B) Aging-related PPI network constructed using STRING database of interactions from [118], C)Angiogenic signaling network, taken from [119].

It has been long recognized, that in living systems, genes and proteins rarely act individually. Indeed, a particular response to environmental stimuli, such as disease, growth, or development is always an integration of multitude of interactions, an intricate web of connections between genes, proteins, and small molecules (Figure 1). Reconstruction, cataloguing, and functional categorization of these interactions is the major goal of “-omics” technologies. Functional understanding of Protein-Protein Interactions (PPIs) and protein-nucleic acid interactions is essential part of describing how a particular genotype yields a corresponding phenotype. Furthermore, having detailed interactional information allows predicting systems behavior in response to a perturbation. For example, interactional information can be used to suggest new drug targets for therapeutic intervention [1–3].

Given its importance, mapping PPI networks has been on proteomics agenda for quite some time: for many organisms the most persistent interactions, either physical, genetic or computationally predicted has been mapped and catalogued. Before the advent of high-throughput technologies, most computational approaches to predict PPIs studied subunit interfaces, employing the information from the protein structure database [4]. The impetus in computational prediction of PPI has been gained by the availability of full genomic information, leading to the appearance of genomic context approaches that consider genome sequences to predict interactions [5]. These approaches use the fact that the genes of functionally interacting proteins are genomically associated with each other. Originally, non-random genomic co-occurrence, such as gene fusion [6,7], the conservation of gene order [8,9] and co-occurrence of genes among sequenced genomes [10] was used to get insights into PPIs.

Majority of the experiments to detect physical PPIs has been relying on Affinity-Purification-Mass-Spectrometry (AP-MS) [11,12] or Yeast-Two-Hybrid (Y2H) [13,14], or Mammalian-Two Hybrid [15] methods. These experiments provided a rich source of protein interactions, deposited in databases such as BioGRID, HPRD, IntAct, DIP and GeneMania [16–20]. Proteome-wide PPIs were collected in a high-throughput screen form a PPI network. In this network, a protein is treated as a node and an indirect link between two proteins (edge) represents physical interaction. This representation re-states the problem of computational prediction of protein interactions, in particular protein complexes, as a well-familiar problem of finding network modules. A module (cluster) in a network is a highly interconnected group of nodes, connected to the nodes outside the group with a few links [21]. The modularity of biological networks was observed for many types of networks [22,23]. It is generally believed that modules tend to represent groups of functionally associated genes/proteins that work together to perform a biological function. In a PPI network functional association refers to a protein complex. Many algorithms have been developed to extract network modules [21,24,25], to identify the functional relationships between nodes and to further predict functional links. It should be noted that the identification of the network modules is possible if the number of edges is to some extent comparable to the number of nodes, otherwise the distribution of edges among nodes is too homogeneous [26]. Several algorithms were developed specifically to identify protein modules-molecular complexes-in a large PPI network (e.g. MCODE [27], RNSC [28], LCMA [29,30]).

Identifying protein complexes from PPI networks requires the high quality of the underlying network. However, the accuracy of high-throughput PPI data is generally low [31], undermined by specific biases, pertinent to different experimental approaches. For example, Y2H interaction networks tend to be enriched for transient interactions [32], while AP-MS tends to map indirect interactions [33]. This is why the algorithms for identifying densely connected PPI network components, although abundant, are not widely used for the inference of protein complexes.

Other experimental methods such as fluorescence resonance energy transfer (FRET [34,35]), and to lesser degree electron spin resonance using spin-labeled pairs [36] has been used to further validate and refine the interaction geometry and protein-protein contacts. Additionally, “hot-spots” of protein-protein interactions - the residues, which stabilize the interaction - has been studied using site-directed mutagenesis approaches (e.g. alanine scanning [37]). The major drawback of these methodsis, perhaps, their large-scale applicability-it takes a lot of effort to map the full interactome using these approaches.

At the same time, it is anticipated that the research demand for fast large-scale network mapping will increase as biologists start to ask questions not just about how different protein levels differ between one condition vs. the other, but how do the interaction networks differ [38,39]. Excellent example of the differential network study is the recent paper by Bisson et al. [40], where the authors examine signaling PPI network, associated with GRB2 adaptor. To map the GRB2-centered PPIs, the authors used a mass-spectrometry approach based on affinity purification of GRB2 and Single-Reaction Monitoring (SRM). Comparing summed SRM intensities of GRB2 interacting partners before and after stimulation of signaling, allowed the authors to quantitate difference in strengths of the interactions. Notably, Bisson et al. [40] focused on the key hub protein, GRB2. However, if we were to pose the question of the network dynamics on the scale of the full interactome, even if focusing only on hubs, it would take considerable effort to get to the answer using the affinity-purification methods.

In this regard, taking a snap-shot of interactome using chemical cross-linking may offer a significant advantage. Potentially, the cross-linking could not only give identities of interacting partners, but it can also provide topology of their interactions in the same setting. Despite being conceptually attractive, chemical cross-linking is notoriously difficult to implement on the large scale. What are the challenges and technological limitations that need to be overcome for the large-scale cross-linking to be an effective PPI research tool? In this review we focus on the following three: 1) In digests of complex protein mixtures, the non-cross-linked peptides always dominate; 2) If the previous problem is solved, say, by enriching for the cross-linked peptides, still, cross-links within the same molecule (intra-protein) will be several folds more abundant than cross-links between different molecules (inter-protein); 3) How does one select a cross-linking reagent to capture most of the interactions, given the high structural and chemical heterogeneity of protein-protein interfaces?

In other words, are the cross-linking chemistry and the current mass spectrometry instrumentation adequate to solve the double ‘needle-in-a-haystack problem’-finding cross-linked peptides amongst non-cross-linked species and discriminating between inter- and intra-cross-links? There are promising studies coming out recently from several proteomics laboratories that point to the positive answer to this question.

## Chemistry of Protein-Protein Interactions

Biologically relevant PPIs are commonly classified into persistent (stable protein complexes) and transient. A given protein may exhibit broad spectrum of affinities to its substrates, which are modulated and regulated by the cell. This is especially evident in the case of transient interactions, which are prevalent during signal transduction processes, where a given protein’s affinity towards its interacting partner is modulated by a certain Post-Translational Modification (PTM). For example, proteins with SH2 domains recognize phosphorylated tyrosines, proteins with bromo-domains recognize acetylated lysines, and proteins with chromo-domains recognize methylated lysines. In principle, by examining physico-chemical properties of known stable and weak protein-protein interfaces it is possible to construct a classifier, which will with reasonable accuracy predict if a given interface is persistent or transient [41,42]. Therefore, in general, the chemistry of interactions is different in transient vs. persistent PPI interfaces.

From the purpose of designing effective cross-linking methodology, which would aim at capturing broad range of transient and persistent interactions, it is also useful to know likelihood that a particular amino-acid or an amino-acid pair is present at a PPI interface. One approach to assess this likelihood is to examine structures of protein complexes deposited into the Protein Data Bank (PDB) and to count amino acid occurrences at the interfaces [43,44]. Table 1 summarizes these findings and shows relative frequencies of aminoacids at protein interfaces. Interestingly, the interface propensity of an amino acid correlates not with its hydrophobicity, but with its propensity to form under-wrapped hydrogen bonds, or “dehydrons”, (Table 1) ([45] for the detailed discussion of the dehydrons). Overall, hydrogen bonds and salt bridges play the most significant role at the interfaces [43], while hydrophobic interaction is the second in the order of importance [46]. On average, a PPI interface has about ten hydrogen bonds, and about two salt bridges [46]. For a random interface there is still a good chance to find amino acids Leu, Ilu, and Val due to their high overall abundance. In the order of decreasing frequency, the following amino acids are over represented at PPI interfaces compared to other surfaces: Asn, Thr, Gly, Ser, Asp, Ala, and Cys [45]. In terms of amino-acid to amino-acid contacts, amino acids pairing with the highest preferences are Cys-Cys, Trp-Pro, Asp-His, Arg-Trp, Asp-Ser, and Asp-Thr [47]. Additional examination of PPI interfaces reveals that structural water and metal ions often participate in the interaction at the interfaces, and often shield charged residues from each other [48].

Further examination of PDB structures of interacting proteins reveals differences in amino-acid composition deep inside the interface (core) and on the periphery (rim). Table 1 summarizes these results in the order of decreasing frequency, residues Lys, Glu, Asp, Pro, Asn, Pro are likely to occur on the rim, while residues Trp, Phe, Cys, His, Met are likely to occur within the core [49]. Importantly, in cores of the interfaces amino acid residues are protected from the outside environment and most likely won’t interact with bulky long-length chemical cross-linkers, especially if the protein-protein interaction is stable. On the other hand, the shorter the length of the cross-linker is, the more likely it will get closer to the interface. In certain cases it is possible to engineer cross-linking groups into the protein itself, which would capture interaction exactly at the interface (e.g. photo-reactive amino acids [50]). In addition, in the case of a transient interaction, there is a possibility that modification with a small, short-length cross-linker prior to the interaction might still capture the interface after the interaction is formed.

It is reasonable to expect long-length cross-linkers to capture interactions away from the interface, while shorter cross-linkers will be capturing the interaction closer to the interface. Also, if we will choose very specific cross-linker, for example zero-length cross-linker 1-ethyl-3-(3-dimethylaminopropyl)-carbodiimide (EDC), which catalyzes condensation of primary amines with carboxylic acids [51,52]; we will obtain only those interactions, which by chance will have the lysines and aspartates/glutamates at the right positions. Consequently, EDC will be blind to all the other interactions. Also, in cases where a carboxylic acid/amine pair is deeply-buried within PPI interface, EDC might not even get to it, thereby ignoring most stable interactions. Yet, at the same time, other interactions that EDC does capture will be close to the PPI interface, because it is a zero-length cross-linker.

In contrast, if we choose a very long, flexible cross-linker coupling a certain amino acid pair there is a good chance that majority of interactions will have at least one such pair at right position within the cross-linker’s reach to be captured. However, it is unlikely that in this scenario, we will get to the PPI interfaces themselves. Another thing to consider is that long-length cross-linker may detect spurious, nonspecific interactions and alter the native protein structure more than the short-length cross-linkers would [52].

We can, therefore, conclude that long-length cross-linkers, selective for frequently occurring amino acids, are good for establishing identities of interacting partners; while shorter, broadly selective cross-linkers are better suited for capturing interacting interfaces themselves.

## Chemistry of Protein-to-Protein Cross-Linking

**Figure 2:**
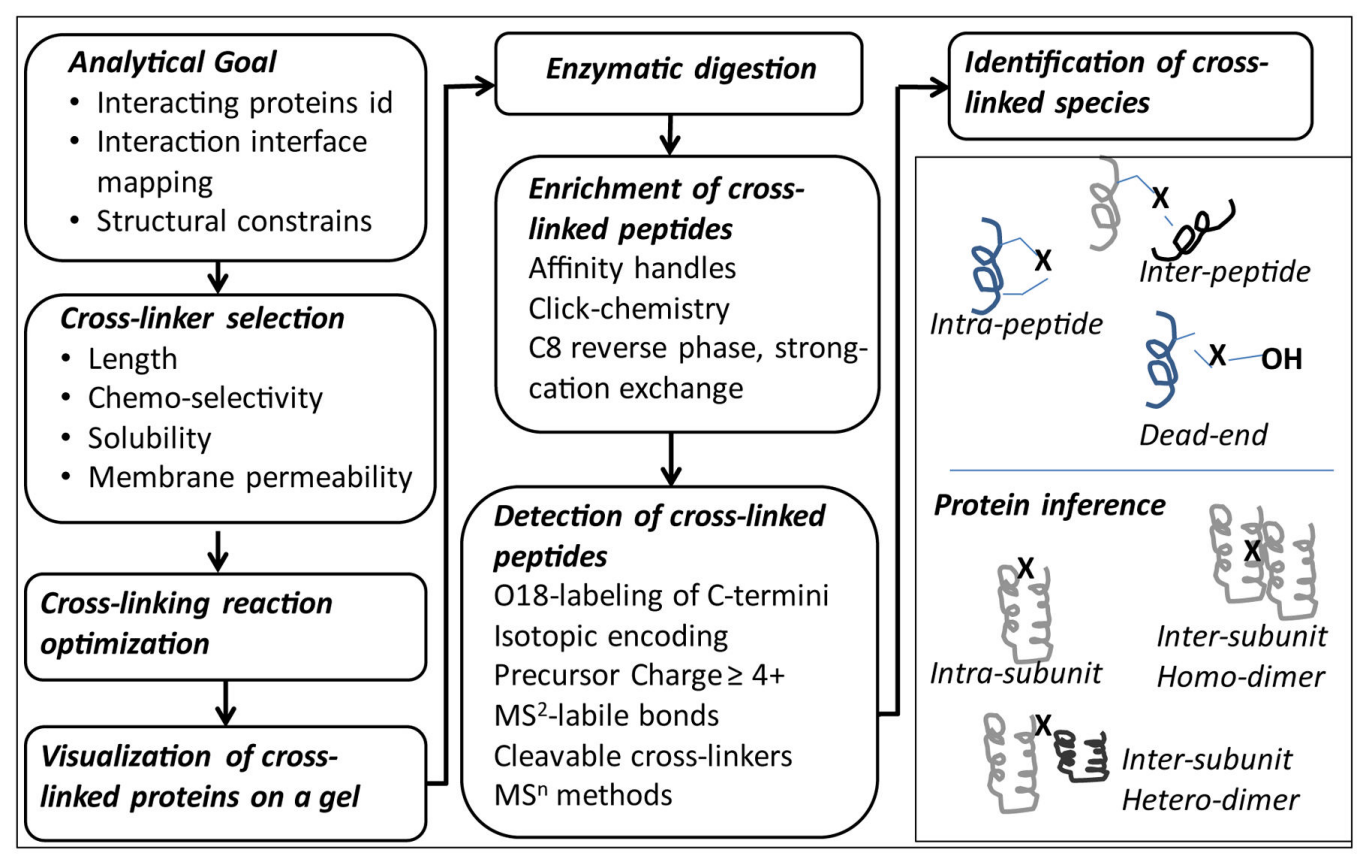
Common steps in a cross-linking mass spectrometry experiment.

**Figure 3:**
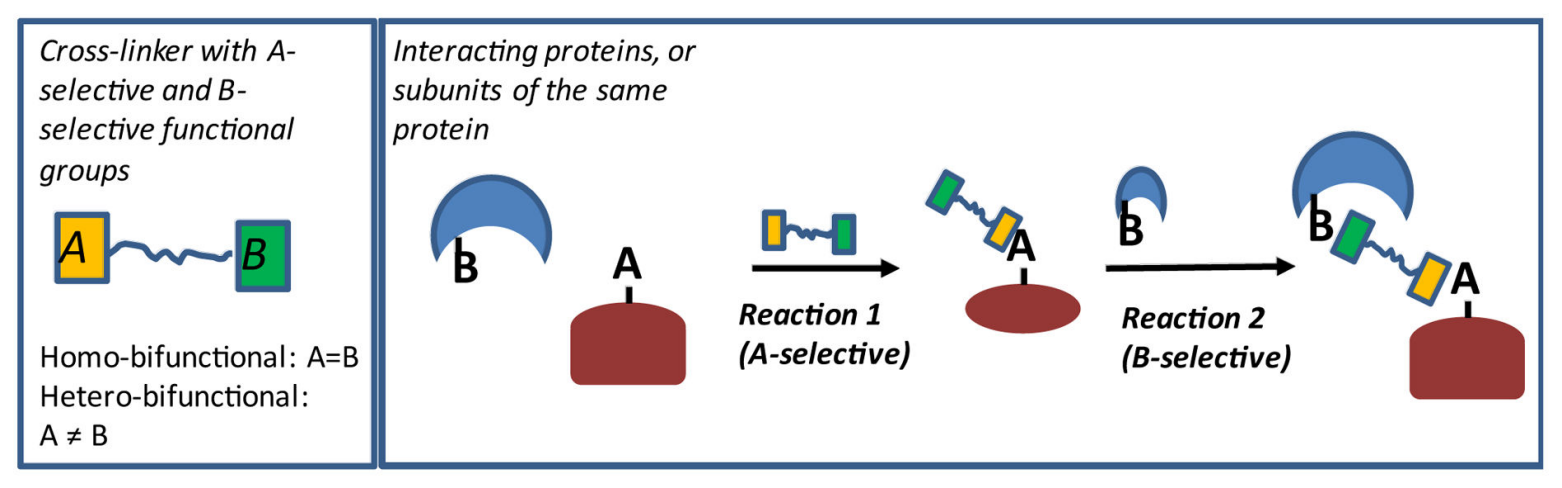
Protein-to-protein cross-linking Cross-linking proceeds in two steps: *Reaction 1* – activation of the first protein. *Reaction 2* – the crosslinking event, which yields ether intra-(same protein) or inter- (different molecule) cross-links.

Figure 2 illustrates common steps undertaken during a typical Cross-Linking Mass Spectrometry (CXMS) study. The exact number and order of the steps depends on the task at hand. Figure 3 provides a general scheme for protein cross-linking. An archetypical cross-linker has at least two functional groups, which are either the same (homo-bifunctional), or different (hetero-bifunctional). Reaction 1 involves activation of the first protein with the first functional group on the cross-linker. Reaction 2 involves cross-linking event itself using the second functional group of the cross-linker. If the reaction 2 takes place within the same protein, the result is called intra-protein cross-link. If it occurs with another protein molecule, it is called inter-protein cross-link. It should be noticed that costs in entropy are different between reactions 1 and 2. For example, if the interaction is formed prior to the cross-linking, and the amino-acids to be linked are at the right geometric positions-the only entropic cost for the reaction 2 is in curbing the rotation of the cross-linker, which was already bound during the reaction 1. In such cases we will expect the chemo-selectivities to be slightly different: even for the homo-bifunctional cross-linkers the reaction 2 will be less selective than the reaction 1. Corollary to the observation of differences in energetics between reaction 1 and 2, is that we may potentially get different results, depending on which protein gets activated first in the reaction 1.

In the following section we will look at what physicochemical properties of cross-linked peptides can be exploited for their detection, and also what functional groups are available for effective cross-linking. We limit our survey to the approaches, which could be potentially useful on the large-scale. For a more detailed discussion, see the recent review by Paramelle et al. [53].

## Physicochemical Properties of Cross-Linked Peptides

A complex protein mixture from a biological specimen is highly heterogeneous, where individual proteins differ considerably in hydrophobicity, molecular weight, and isoelectric point. Enzymatic digestion, while resulting in increased number of distinct molecules (peptides derived from the starting proteins), shrinks the physicochemical space, thereby simplifying peptide detection, peptide identification, and protein inference. This is the basis for “bottom-up” protein identification experiment, where each of the protein from the starting protein mixture can be identified by several different peptides [54].

In a digest by trypsin protease, which cuts after Lys and Arg residues, peptides on average are around 12–15 amino acid in length, with majority having charge 2^+^ at pH 2.5 (which is usual pH during ionization/mass spectrometry in a bottom-up proteomics experiment). One of the two charges is due to protonation of peptide N-terminus, and the other one is due to protonation of the C-terminal Lys or Arg. Small proportion of lower charges is due to non-specific cleavages, protein C-termini, and occasional loss of a proton during ionization process. Similarly, small proportion of higher charges is due to incomplete digestion, His residues, and occasional capture of a proton during ionization process.

When using non-specific protease (e.g. proteinase K), one could manipulate reaction times and temperature to produce peptides of desired average length [55]. In this case, however, because of the absence of the selection for Lys- and Arg-containing peptides, higher proportion of lower charges is expected for the shorter peptides.

Next, when the enzymatic digestion of cross-linked proteins occurs, we can expect the cross-linked peptides to be different from the non-cross-linked ones in the following properties: i) charge to be doubled on average; ii) length to be doubled on average; iii) cross-linked species to have at least two C-terminal carboxy- groups and two N-terminal amino-groups. Noticeably, the properties i-iii) are not dependent of the nature of the cross-links. It is, therefore, advisable to use these properties for the large-scale studies when employing shortlength, zero-length, or natural cross-links.

Enrichment by strong-cation exchange resin [56,57] (increase in charge), or using size exclusion chromatography [57] (increase in size) have been used as effective cross-link enrichment strategies. On a side note, the authors [57] also showed that the use of Asp-N protease parallel to trypsin boosted the number of detectable cross-links. Detection of highly charged cross-linked peptides is facilitated further by the high resolution mass spectrometry, available on modern hybrid instruments [58]. With the high resolution, charge of a peptide can be unambiguously assigned based on the separation of its isotopic peaks on the m/z axis, thereby enabling charge-driven acquisition of the fragmentation spectra. This strategy has been proved very effective to detect the cross-linked peptides [59].

The third property-increase in the number of carboxy and N-termini within cross-linked peptides can be exploited via selective chemical labeling. Digestion by trypsin in O^18^ water, which introduces two O^18^ atoms into a peptide C-terminus, has been used in quantitative proteomics for a long time. In a typical O^18^ quantitation experiment, one sample is digested in O^18^ and another in O^16^ followed by 1:1 mixing. Because the chromatographic properties are not affected, O^18^ and O^16^ peptides co-elute and enter mass detector at the same time, with O^18^:O^16^ ratio indicative of relative abundance of the protein in the mixture [60,61]. When applied to the cross-linked samples, one half of the sample is digested in O^18^ and another in O^16^ water. Next, the samples can be analyzed either after mixing, or separately [62,63]. As a result, the cross-linked peptide pairs will be separated by ~8 Da (two labeled C-termini), while non-cross-linked peptides, will be separated by ~4 Da (one labeled C-termini). Using this approach one also needs to be aware of the additional incorporation of O^18^ atoms during non-enzymatic deamidation of Asn and Gln [64–66] as well as back-exchange [67] and incomplete labeling [68,69]. Similar to the counting of C-termini using incorporation of O^18^, albeit less frequently used, N-termini also can be counted by post-digestion chemical modifications [70]. In fact, the authors of [70] demonstrated that if a 1:1 heavy:light mixture is used to modify N-termini, inter-peptide crosslinks exhibit a distinct isotopic signature (a 1:2:1 ratio). Just like with the deamidation of asparagines producing artifacts in the O^18^ method, here, special attention needs to be paid to miss-cleaved lysines residues and reduction in charge.

It would be interesting to combine the N-terminal and C-terminal chemical labeling of cross-linked peptides; as we anticipate that such methods could be particularly useful for large-scale differential PPI studies. Recently, Zhang et al. [71] reported successful use of similar strategy for analysis of N-glycosylation sites. The authors combined an N-terminus-labeling reagent, isobaric tag for relative and absolute quantitation (iTRAQ) [71–73] with O^18^ labeling to quantify the glycosylated and non-glycosylated peptides. The glycosylation sites were marked via PNGase F catalyzed labeling in O^18^/O^16^ water. Using the multiplexing capability of the iTRAQ reagents, the authors performed simultaneous analysis of four samples.

The above methods can be employed on the large scale to improve detection of any type of protein-to-protein cross-links, including short-length and zero-length. For longer cross-linkers, on the case-by-case basis, their chemical structure can be manipulated further to enhance enrichment, detection, and analysis of the cross-linked species. These strategies include isotopic coding, affinity handles, click-chemistry handles, ionization enhancers, reporter ions, and MS^2^-labile bonds [53].

In the following sections we will examine functional groups on commercial and custom-synthesized cross-linkers, which could be potentially useful in large-scale studies.

## Amine-Reactive Functional Groups

**Figure 4:**
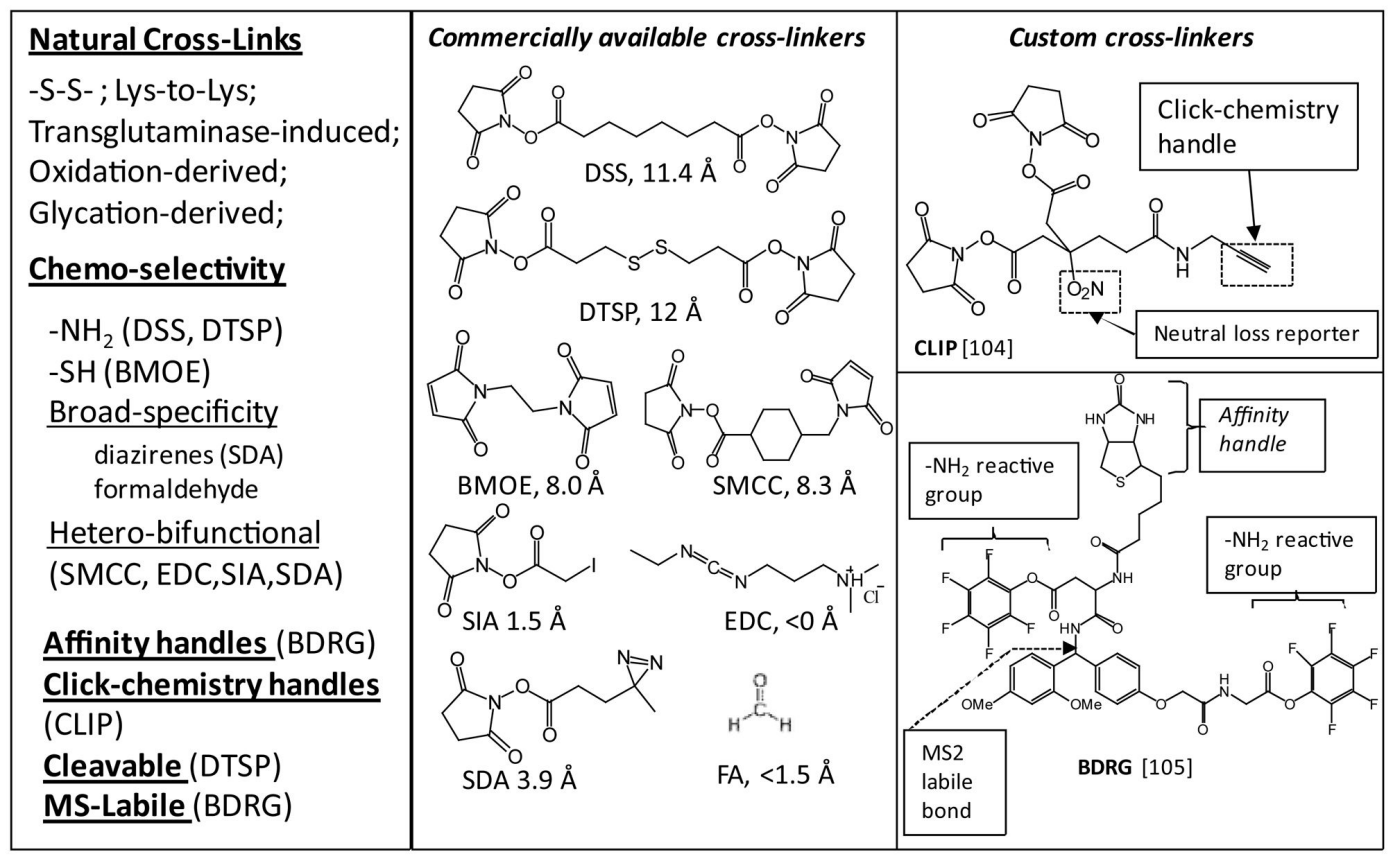
Cross-linking reagents commonly used in PPI studies Cross-linker selection: depending on the analytical task at hand, a cross-linker is chosen based on its length and physico-chemical properties (left panel). A variety of cross-linking reagents are commercially available (middle panel). Custom-synthesized crosslinkers include MS-labile groups, affinity handles, and conjugation-chemistry handles for facilitating detection and purification are shown in the right panels.

N-hydroxysuccinimidyl-ester (NHS-ester) is the most popular functionality in this category. Perhaps, the most common cross-linking reagents - DSS (Figure 4) and its soluble analog BS3 are used in various types of cross-linking experiments to yield irreversible cross-links. In addition to primary amines, lysine side chains and protein N-termini-NHS group has some reactivity towards serine, threonine, and tyrosine [74], especially at the second step of the cross-linking for the entropic reasons already discussed. Overall, the DSS cross-linking reaction is reasonably fast and selective, giving accurate snap-shot of PPIs present in solution. Cross-link introduced by DSS is 11.3 Å long, but shorter and longer versions of homo-bifunctional NSH-esters are also available; including cleavable reagents and molecules with Polyethylene Glycol (PEG) linkers to increase the reagent’s solubility.

Recently, other amine-reactive functional groups -N-hydroxyphthalimide, hydroxybenzotriazole, and 1-hydroxy- 7-azabenzotriazole have been shown to have faster reaction times and improved efficiency compared to DSS [75]. Imido-esters and thioimido-esters another promising functional group for the amine-to-amine cross-linking studies. Advantage of these reagents over DSS is that they preserve positive charge, thereby minimizing alteration of native protein structures [76]. In addition, the higher charges often imply easier enrichment and detection, and in many cases more complete fragmentation in tandem MS [77].

Because lysine is quite abundant amino acid residue (Table 1), the use of amine-to-amine cross-linkers is quite effective in variety of applications. For example, variable-length amine-to-amine cross-linkers are often used to constrain the Lys-to-Lys distances. Even though these constrains provide rather weak structural information, using cross-links in conjunction with molecular modeling is usually sufficient to determine the structure of a protein complex or single protein at a moderate resolution [78–80]. There are also several studies, which use DSS type cross-linkers on large scale. For example, Rinner et al. [81] used *E. coli* whole-lysate cross-linking to demonstrate utility of the xQuest algorithm. To enable the large-scale analysis the authors employed isotopically coded DSS cross-linker (DSS-d0/d12 pair) and focused on highly charged precursors. Majority (above 3000), of the detected cross-links were intra-protein, while only 71 were inter-protein cross-links, which included subunits of Serine hydroxymethyltransferase (GLYA), GroEL, Tryptophanase (TNAA) and the small ribosomal subunit. The authors further validated most of these inter-protein *E. coli* cross-links by examining corresponding X-ray structures.

Similarly, in the more recent analysis by Yang et al. [82], introducing the software pLINK, using BSS-d0/d4 isotopically coded cross-linking and high-resolution High-energy Collision Dissociation (HCD), identified 394 interlinks from *E. coli* lysates.

## Sulfhydryl-Reactive Functional Groups

Alkyl iodides and maleimides [83], and less often, thiopyridines [84] are used to selectively modify cysteine residues in proteins. Homo-bifuctionalbis-maleimido cross-linkers are often employed on a small sale for the Cys-Cys coupling [85]. Because Cys residue is rarer compared to Lys (Table 1), sulfhydyl-reactive functionality may be less useful in large-scale PPI studies. In a hetero-bifunctional format, e.g. NHS-Maleimide, Lys to Cys cross-linking has its uses as a structural technique to derive Lys-Cys distance constrains [86]. Succiminidyliodoacetate (SIA) is a short-length (~1.5 Å) hetero-bifunctional cross-linker, which could be potentially useful for mapping of rims of PPI interfaces.

## Hydroxyl-Reactive Functional Groups

Isocyanate moiety, -NCO, is used for the purpose of hydroxyl-specific reaction, but is rarely used in the PPI studies. This is probably because the -NCO reacts well with stronger nucleophiles-primary amines and sulfhydryls. It does require un-protonated amine for the reaction, therefore, at physiological pH this leaves threonines, serines, and histidines as likely sites of the –NCO action. Another reason for the limited use of the –NCO chemistry is that the isocyanate coupling is a rather slow reaction; and for effective cross-linking faster reaction times are preferred. However, the activated –OH groups, such as in the active sites of serine proteases, are very reactive towards –NCO [87]. In fact, this is one application where –NCO cross-linking chemistry could be useful-selective cross-linking of serine proteases with their substrates.

## Carboxyl-Reactive Groups

Carbodiimides, such as already discussed EDC, activate carboxyl groups for condensation with primary amines via amide bond formation [88,89]. We are not aware of much other carboxyl-selective functionality used in chemical cross-linking of carboxyl groups. One other possibility is dehydration induced carboxy-to-amino condensation in the solid state [88]. Potentially the latter approach could be more useful in capturing amino-to-carboxy ionic bridges deep inside stable PPI cores, which EDC would ignore.

## Photo-Reactive Groups

Photo-reactive groups which are used in many types of bioconjugation including protein cross-linking, are diazirines [90], azides [91], and benzophenones [92] (Figure 4). Upon photolysis, these groups generate highly reactive free radical species, which show almost no chemo-selectivity. Typically, the photo-reactive functionalities are used in hetero-bifunctional cross-linkers that are highly chemo-selective in the reaction 1, while the non-selective photo-reactive moiety is employed in the reaction 2. NHS-diazirine cross-linkers (Figure 4, SDA) are of this variety. We are not aware of any large-scale study using such cross-linking reagents. On the smaller scale, however, SDA was proved highly effective. For example, Gomes and Gozzo studied cross-linking of model peptides and equine myoglobin using 13.5 Å long, cleavable NHS-diazirine cross-linker, SDAD [93]. As expected, the NHS-diazirine generated higher number of cross-links, compared to DSS. Also, using MALDI-MS/MS and ESI-qTOF-MS/MS platforms, the authors showed that all cross-linked spectra had characteristic ions indicative of carbene insertion.

We therefore expect that when used on the large-scale the sheer number of captured interactions with NHS-diazirine cross-linkers should be higher than those obtained with Lys-to-Lys cross-linking. Moreover, the existence of predictable fragmentation of the points of carbene insertion as demonstrated [93] can aid in the cross-link identification.

Photo-active Leu and Met analogues containing diazirine groups are especially interesting, as Leu and Met are very abundant (Table 1). Suchanek et al. [50] demonstrated that these amino-acids can be incorporated into proteins in live cells by native cellular translation machinery. Using this method, Suchanek et al. [50] discovered a novel interaction of the progesterone-binding membrane protein PGRMC1 with Insig-1, a regulator of cholesterol homeostasis.

Other genetically encoded un-natural amino acids, which have photo-activable functional groups has also been used to explore protein-protein interactions [94–97].

## Formaldehyde

Arguably, formaldehyde (FA) is the most important cross-linker. It is frequently used on the large scale to fix, conjugate, and crosslink proteins and protein-to-nucleic acids. FA has been shown to aid in affinity purification; stabilizing specific interactions within protein complexes [98]. There are numerous tissue banks, which are filled with Paraffin-Embedded Formalin-Fixed (FFPE) samples that potentially hold therapeutically valuable PPI information. FA reaction times are fast; FA easily penetrates membranes, and therefore is next-to-ideal cross-linker for *in vivo* studies. It is also easily reversible at elevated temperatures.

Generally, FA is considered a broad-specificity cross-linker and has potential to cross-link any nucleophile to any nucleophile in a protein. In practice, reaction 1 proceeds the fastest with primary amines, and the reaction 2 with primary amines, histidines, asparagines, glutamines, tyrosines, and arginines [99]. The reaction products are quite heterogeneous in context, and structure-dependent [100] and, in addition to simple methylene bridges, the cyclic and polymeric end-products are abundant. It is therefore important to optimize the FA-cross-linking conditions to maximize the benefit. If more selective cross-linking is desired, reaction times should be short, and formaldehyde concentration low. In contrast, if broader specificity is desired, and there is not much concern about the ill-defined products of formaldehyde modification, reaction times could be longer. Concentration range at 0.1–2% is a typical range for the PPI studies using FA. Low FA concentrations (0.1–0.5%) are also more selective and yield mostly Lys- and Trp-directed cross-links [99].

The FA’s broad specificity and chemical diversity of the end products complicates MS-based proteomics analysis. Despite such difficulties, successful strategies for FA cross-link identification are advancing, and we anticipate seeing more such studies in the future [101,102].

## Multi-Functional Cross-Linker Design

For the purposes of enrichment and facilitation of cross-link detection during data acquisition, variety of other functionalities in addition to chemo-selective groups has been explored for the design of cross-linkers. Most frequent are click-chemistry handles, biotin handles, reporter ions and fragmentation specific cleavages [53]. Click-chemistry, i.e. copper-catalyzed alkyne-azide conjugation [103], is an attractive cross-link enrichment strategy for the large scale studies because the reaction is very fast and selective; plus, alkyne handle itself is compact and inert.

Chowdhury et al. [104] demonstrated effectiveness of this approach in the form of a novel Lys-to-Lys cross-linker ((Figure 4), CLIP). The CLIP cross-linker has alkyne group for the click-chemistry capture, and the reporter-NO^2^ group, which allows for neutral loss scanning and also increases water solubility of the reagent. The authors further evaluated the click-chemistry effectiveness in enriching cross-linked proteins from complex backgrounds, by mixing the CLIP-cross-linked samples with *E. coli* lysates. As a result, for as low as 1:100 mixing into the non-cross-linked protein background, enrichment efficiency of the cross-linked peptides remained exceptionally high. Importantly, CLIP has the same chemo-selectivity as DSS, and is almost the same length. We, therefore, anticipate that CLIP can easily replace DSS in large-scale cross-linking and structure analysis, because in comparison to DSS; enrichment yields more interactions and provides higher quality mass spectrometric evidence.

In a similar study, using biotin-handle along with MS^2^-labile bond engineered into the cross-linker ((Figure 4), BDRG), Luo et al. [105] demonstrated effectiveness of this strategy on the scale of large protein complexes. Luo et al. [105] used the MS^2^-labile bond, and the subsequent MS^3^ scans of resultant fragments to enable rapid identification of the cross-linked peptides using regular database search.

In the latter study the MS^2^-labile bond is called Rink bond; due to the stability of its fragmentation products this bond is more labile than peptide bonds. One could also use two Rink functionalities in the same molecule, so that cross-linker is cleaved out during fragmentation process and serves as a reporter. Such strategy was implemented by Zhang et al. [106] using novel cross-linker called Protein Interaction Reporter (PIR). PIR has also biotin handle in addition to the two Rink groups for the enrichment of cross-linked proteins. Zhang et al. [106] also studied another MS^2^ mobile bond - Asp-Pro - as an alternative to Rink. The authors applied the PIR method to *Shewanella oneidensis* bacteria and showed that PIR is a particularly well suited for capturing interactions of membrane proteins.

## Disulfide Bonds

While being rare, Cys is enriched at PPI interfaces (Table 1). Plus, whenever it does occur in PPI interface it is most likely to form disulfide bond with another Cys from the interacting protein [47]. Therefore, it is beneficial to map disulfide bridges within large scale studies of PPIs, in conjunction with other approaches, in order to increase number of captured interactions.

Because disulfide bonds are easily reduced, overlaying LC-MS maps in reduced vs. oxidized samples allows for the cross-link detection. Peptides originally carrying a disulfide bond are recognized due to the shift in both retention time and m/z values, whereas peptides containing no cysteine stay the same. Such approach was undertaken by Evaristo et al. [107] in the identification of disulfide bridges within skin venom of several amphibian species.

Another approach for the disulfide-specific detection is to exploit the disulfide affinity for electrons in the gas phase. Fragmentation techniques, such as Electron-Transfer Dissociation (ETD) and Electron-Capture Dissociation (ECD), can be used to fragment crosslinked peptides selectively at the disulfide bond [108]. Next, performing MS^3^ on the resulting fragments allows for accurate identification of the cross-linked sequences [109]. Ultraviolet irradiation in the gas phase also has been reported to selectively cleave the disulfide bonds [110].

## Cross-Link Identification Algorithms

For a small-scale analysis, it is relatively easy to design a crosslink search algorithm, which uses a database consisting of pair-wise combination of peptides from interacting proteins. Next, this database is used to constrain masses of possible cross-linked precursors. Examples of such algorithms are abundant: CLPM [111], xComb [112], GPMAW [113], X!Link [114], StavroX [98], MassMatrix[115].

Within the Massmatrix it is also possible to use multi-staged strategy, which can be applicable on large-scale [115]. Notably, crosslinking analysis is not the primary function of MassMatrix, which is really a complex proteomics platform, including stand-alone database search engine with parallel computation capabilities, use of multiple fragmentation modes, and different quantitation strategies. The multi-stage implies running regular protein identification search first, and limiting the subsequent cross-links search to reliably identified proteins.

For complex protein mixtures the database of peptide-pairs becomes impractically large and different approaches are required. Generally, we need software platforms, which could use information from isotopic coding, O^18^-labeling, reporter ions, and MS^3^ experiments to reliably detect cross-linked species. Additionally, we need algorithms incorporating *de-novo* sequencing, open-search modifications strategies, high-resolution mass spectrometry and multiple fragmentation modes to reliably identify the cross-linked species. Software platforms such as pLINK [82], xQuest [81], XLink- Identifier [116] are in this category. Cross-link identification pipe-lines using existing algorithms for open modification search [59] and *de novo* sequencing are of particular interest. Indeed, as already discussed, while the use of non-specific cross-linker’s groups, such diazirines may complicate the bioinformatics analysis, it is exceptionally attractive to use on the large-scale; and *de novo* approaches could be the key technology here.

## Conclusion

In summary, we suggest that the short-length, broad-specificity cross-linkers are the most suited for the large-scale studies, including cross-linking in live cells. In addition, it is always useful to perform the disulfide mapping, as Cys-Cys coupling is frequent at the PPI interfaces. Therefore, methods, which exploit most general physicochemical properties of cross-linked peptides-higher charge, bigger size, and higher number of C-termini, are the most suited for the large-scale analysis. At the same time, complementary analysis with specific, long-length cross-linkers will be desirable for the validation and sensitivity-evaluation purposes. The current stage of technology-high resolution mass spectrometry, hybrid instruments, methods of multidimensional separation, multi-stage fragmentation techniques-is adequate, in our opinion for the large-scale cross-linking. Amongst the three main obstacles listed in the introduction, identifying inter-protein cross-links in the background of intra-protein cross-links is the most general and difficult problem to solve. The bigger dynamic range of detection and the shorter scan times become on the modern mass spectrometers, the more inter-protein cross-links will be identified in the large-scale PPI studies. However, in the case protein of homo-dimers and oligomers without available X-ray structure novel approaches to validate intermolecular cross-links will be needed.

## Figures and Tables

**Table 1:** Amino acid frequencies within protein-protein interfaces.

Amino Acid	Interface Propensity [45]	Total Abundance [45]	Rim/Core frequency [49]	Dehydron Propensity [45]	Hydropathy
**Asn**	1.28	3.36	1.19	1.63	−3.50
**Thr**	1.10	4.87	1.19	1.41	−0.70
**Gly**	0.99	7.30	1.16	1.42	−0.40
**Ser**	0.60	4.66	1.04	0.80	−0.80
**Asp**	0.34	5.42	1.48	0.76	−3.50
**Ala**	0.29	7.77	0.95	0.60	1.80
**Cys**	0.25	0.78	0.45	0.24	2.50
**Val**	0.20	8.17	1.09	−0.31	4.20
**Met**	0.10	3.00	0.67	0.10	1.90
**Tyr**	0.10	2.41	0.54	0.10	−1.30
**His**	−0.25	4.35	1.24	−0.25	−3.20
**Pro**	−0.25	1.92	0.52	−0.25	−1.60
**Trp**	−0.33	1.02	0.32	−0.40	−0.90
**Arg**	−0.35	8.91	0.82	−0.40	−4.50
**Leu**	−0.35	6.27	1.19	−1.10	3.80
**Phe**	−0.40	3.61	0.33	−0.40	2.80
**Lys**	−0.42	7.76	2.16	−0.38	−3.90
**Glu**	−0.50	8.59	1.87	−0.11	−3.50
**Gln**	−0.62	3.15	1.03	−0.60	−3.50
**Ile**	−0.70	6.66	0.76	−0.92	4.50

## Abbreviations

PPI: Protein-Protein Interaction
AP-MS: Affinity Purification Mass Spectrometry
Y2H: Yeast Two Hybrid
SRM: Single-Reaction Monitoring
PTM: Post-Translational Modification
SH2: Src Homology 2
PDB: Protein Data Bank
FRET: Fluorescence Resonance Energy Transfer
EDC: 1-Ethyl-3-(3-Dimethylaminopropyl)-Carbodiimide
CXMS: Cross-Linking Mass Spectrometry
iTRAQ: Isobaric Tag for Relative and Absolute Quantitation
MS^2^: Tandem Mass-Spectrometry, or recording of a fragmentation spectrum of a molecule
MS^3^: MS-to-the-third, or recording of a fragmentation spectrum of a peak from MS^2^
NHS: N-Hydroxysuccinimidyl
PEG: Polyethylene Glycol
HCD: High Energy Collision Dissociation
SIA: Succiminidyliodoacetate
SDA: Succinimidyl-Diazirine
SDAD: NHS-SS-Diazirine
DSS: Disucciminidylsuberate
BS3: Bissulfosuccinimidylsuberate
MALDI: Matrix-Assisted Laser Desorption Ionization
ESI: Electrospray Ionization
qTOF: quadrupole Time-of-Flight
FA: Formaldehyde
FFPE: Paraffin-Embedded Formalin-Fixed
CLIP: Click-Enabled Linker for Interacting Proteins
BDRG: Biotin-Aspartate-Rink-Glycine
PIR: Protein Interaction Reporter
ETD: Electron-Transfer Dissociation
ECD: Electron-Capture Dissociation
